# Professional mathematicians do not differ from others in the symbolic numerical distance and size effects

**DOI:** 10.1038/s41598-020-68202-z

**Published:** 2020-07-13

**Authors:** Mateusz Hohol, Klaus Willmes, Edward Nęcka, Bartosz Brożek, Hans-Christoph Nuerk, Krzysztof Cipora

**Affiliations:** 1 Copernicus Center for Interdisciplinary Studies, Jagiellonian University, Krakow, Poland; 2 Clinic of Neurology, University Hospital, RWTH Aachen University, Aachen, Germany; 3 Institute of Psychology, Jagiellonian University, Krakow, Poland; 4 Department of Psychology, University of Tübingen, Tübingen, Germany; 5 LEAD Graduate School and Research Network, University of Tübingen, Tübingen, Germany; 6 Centre for Mathematical Cognition, Loughborough University, Loughborough, UK

**Keywords:** Psychology, Human behaviour

## Abstract

The numerical distance effect (it is easier to compare numbers that are further apart) and size effect (for a constant distance, it is easier to compare smaller numbers) characterize symbolic number processing. However, evidence for a relationship between these two basic phenomena and more complex mathematical skills is mixed. Previously this relationship has only been studied in participants with normal or poor mathematical skills, not in mathematicians. Furthermore, the prevalence of these effects at the individual level is not known. Here we compared professional mathematicians, engineers, social scientists, and a reference group using the symbolic magnitude classification task with single-digit Arabic numbers. The groups did not differ with respect to symbolic numerical distance and size effects in either frequentist or Bayesian analyses. Moreover, we looked at their prevalence at the individual level using the bootstrapping method: while a reliable numerical distance effect was present in almost all participants, the prevalence of a reliable numerical size effect was much lower. Again, prevalence did not differ between groups. In summary, the phenomena were neither more pronounced nor more prevalent in mathematicians, suggesting that extremely high mathematical skills neither rely on nor have special consequences for analogue processing of symbolic numerical magnitudes.

## Introduction

Numerical knowledge is encoded in multiple formats serving specific functions^1–3^. The first kind of code contains the analogue representation of number magnitude; the second one encompasses the visual form of numbers; and the third one stores linguistic representations of numbers. Regarding the first code, namely, analogue magnitude, there is a large body of evidence for shared behavioural characteristics of comparative judgements on symbolic numbers, e.g., Arabic^4–6^, non-symbolic numerals, e.g., sets of dots^7,8^, and other continua including line length^9^, angle^10^, physical object size^11,12^, luminance^13,14^, and non-directly perceivable properties like intelligence^15,16^. Walsh^17^ proposed the “theory of magnitude” (ATOM) for the processing of these and other continua, which can be thought of in terms of classification of “more or less than.”

### Characteristics of analogue numerical magnitude processing

Analogue magnitude comparisons have been studied in different human cultures, languages and notations^7,18,19^, as well as age groups^20,21^. Moreover, there is an extensive knowledge base regarding magnitude comparisons in various non-human animal species^22–24^, from insects^25^, through fish^26^, amphibians^27^, and birds^28^, up to monkeys^29^ and apes^30^. Taken together, these studies suggest presence of the analogue numerical magnitude representation among human beings and its deep evolutionary origins^31^. On the other hand, studying numerical magnitude comparisons in animals is basically limited to non-symbolic material. Analogue magnitude comparisons, both involving symbolic and non-symbolic numerical instances, are often assumed to be driven by Weber’s law and performed by a single cognitive system called the Approximate Number System (ANS)^19,32^. However, other approaches have also been proposed^33,34^. One of them states that non-symbolic comparisons are handled by the ANS, while symbolic ones are served by the Discrete Semantic System (DSS) resembling the mental lexicon^34^.

Despite the analogue magnitude representation having been intensely studied in non-symbolic format, it also has a prominent role in symbolic number processing. Several models of symbolic number processing consider it as an important component^1,4,35^. The analogue magnitude representation underlying processing of *symbolic* numbers is the main focus of this study. The numerical distance effect (NDE) is one of the fundamental characteristics of analogue magnitude processing^4,36^. In case of the comparison of two numbers, performance is poorer (i.e., reaction times, henceforth RTs, are longer, and accuracy is lower) for numbers that are closer together (e.g., 4 and 5) than for ones that are farther apart (e.g., 1 and 5). The numerical size effect (NSE) is another manifestation of analogue magnitude processing^37^. For an identical numerical distance, performance is better when numbers are small (e.g., 1 and 5) than when they are large (e.g., 5 and 9). These effects were initially found for symbolic number processing^4^ and later for non-symbolic numerosities^7^. Both effects can be well observed in tasks comprising judgments of single-digit numbers. Importantly, such tasks are less loaded with strategy use and domain-general processes, as compared to tasks using multi-digit numbers^38–41^. Here we are interested in the analogue magnitude processing of symbolic numbers. Thus, unless otherwise stated, we use the abbreviation NDE referring to the symbolic NDE. The same applies to the NSE.

The characteristics of basic analogue processing of symbolic numbers have been traditionally elucidated by Restle’s^5^ metaphor of the mental number line, where representations of numbers are organized as points in a spatial structure with larger distances between small numbers and smaller distances between large numbers (logarithmic compression)^19,32^ or a more diffuse representation of large numbers. On the other hand, the NDE and the NSE can be accounted for without any recourse to spatial mapping^42^. Magnitude can be activated independently from its spatial association^43^.

Some authors argue that phenomena quantified by the NDE and the NSE can also be accounted for by the numerical ratio effect. As the ratio effect depends on both the numerical distance between numbers to be compared and their absolute magnitudes, it considers both the NDE and the NSE. Performance decreases (longer RTs, higher error rates) with increasing numerical ratio. However, considering the ratio does not allow to investigate the NDE and the NSE separately. Research on the numerical ratio effect gained more popularity in studies investigating non-symbolic number processing^44^. Nevertheless, some studies used it to account for symbolic number processing effects^45^ as well. Therefore, in addition to calculating the NDE and the NSE, we also studied ratio effects (see Supplementary Material 1 for more detail).

### Basic numerical processing and mathematical skills

It is often argued theoretically that basic numerical processing serves as a scaffold for the acquisition of arithmetic concepts and full-fledged mathematical competences in general^19,32,46,47^. Investigating how characteristics of basic numerical processing actually relate to more advanced mathematical skills is one of the crucial aims of the differential psychology of mathematical cognition^48,49^. The idea is that more complex mathematical cognition cannot develop properly if the basic representations on which mathematics is based, are not properly built. However, evidence for such relationships between basic numerical representations and complex mathematical skills is mixed and differs largely depending on the signature of basic number processing under scrutiny. The performance on several tasks tackling some representations correlates positively with mathematical achievement, but other tasks reveal no correlation or somewhat mixed evidence^50–52^. Moreover, even different variants of the tasks considered to engage the same cognitive system could correlate with mathematical skills in different ways, depending on the stimuli. In particular, the association between numerical magnitude processing and mathematical achievement is stronger in magnitude tasks involving symbolic numbers in comparison to non-symbolic magnitude tasks^53,54^. Therefore, we decided to focus on symbolic number processing. However, this does not preclude incremental variance to be explained by non-symbolic processing (for reviews on relations between non-symbolic number processing and mathematical skills see^48,52,55^).

### Symbolic numerical distance effect and mathematical skills

The analogue numerical magnitude representation is the most ubiquitous fundamental representation of numbers in the cognitive system. Its hallmark manifestation is the NDE. It is assumed that when the analogue numerical magnitude representation is precise and refined, the NDE should be smaller^52,56^. Conversely, a larger NDE seems to be associated with a more imprecise analogue magnitude representation.

Although the analogue numerical magnitude representation and the NDE constitute a fundamental representation and its manifestation respectively, studies show a somewhat inconsistent picture of relationships between the NDE and mathematical skills. Early studies demonstrated that the NDE decreases with age during childhood and stabilizes around the fourth grade^57^. This suggests that the NDE reflects a numerical representation which changes and gets refined during normal development. It is worth noting that this observation has been questioned because the NDE can be driven by changes in general RT pattern, and it is known that RTs become faster and less variable with age. Additionally, opposite effects can be found if effect size is considered rather than raw RT^45^. Nevertheless, despite its size changes with age, the NDE remains robust in adulthood. The results of several studies on groups with typical mathematical skills levels did not provide clearcut results: usually, the size of the NDE does not explain a substantial amount of variance in mathematical skills^53,58^. On the other hand, mathematical skills correlate at a moderate level with overall RT (i.e., not the NDE) in the magnitude classification task^59^, which may be indicative of easiness of access to numerical magnitude in general.

Studies on participant groups with mathematical difficulties have also provided inconclusive results ranging from a larger NDE^60–62^ through no differences in the NDE^63^ to a smaller NDE^64^ when compared to groups without mathematical difficulties. There are also case reports of a reverse NDE in dyscalculic individuals^64,65^. The NDE was also observed in a calculation prodigy^66^.

It seems that there is no genuinely strong and consistent relationship between the NDE and mathematical skills level when groups with typical mathematical abilities and mathematical difficulties are taken into account. However, all of these studies compared only participants with poor mathematical skills to a control group. Except for the one report of a calculation prodigy, groups displaying extremely high mathematical skills levels are largely understudied and to the best of our knowledge, the latter groups have not been systematically tested as regards the NDE. Professional mathematicians may differ from other groups in terms of their NDE. For instance, they differ from controls in their SNARC effect^67^ (in the parity judgement task with symbolic numbers) and positive number mapping (in the number line task)^68^; thus, differences in basic numerical effects are possible. Such differences could be due to intense training and exposure to numbers leading to a more precise analogue magnitude representation, or because possessing a specific type of magnitude representation fosters the mastery of professional mathematical skills and thus helps one to become a professional mathematician.

### Symbolic numerical size effect and mathematical skills

Compared to the NDE, our knowledge on the relationship between the size of the NSE and mathematical skills is considerably weaker. As Rousselle and Noel^64^ reported, the NSE is reduced, similarly to the NDE, in children with mathematical learning difficulties as compared to children without difficulties. On the other hand, Núñez-Peña and Suárez-Pellicioni^69^ found that highly math-anxious individuals revealed a larger NSE (the same is true regarding the NDE) than less math-anxious participants, suggesting that the first group is characterized by less precise access to numerical magnitude. In summary, although some studies report differences in the NSE and the NDE related to mathematical skills, no consistent picture has emerged yet.

### Individual prevalence of symbolic numerical distance and size effects

As we have already mentioned, the NDE and the NSE are considered to be highly widespread among human cultures and age groups, yet little is known about their frequency of occurrence at the individual level. Recently, a distinction of group level psychological phenomena into dominant and indominant ones has been proposed^70^. Dominant phenomena are present in virtually all individuals and no one reveals a divergent effect. Indominant phenomena are present only in some individuals, and some individuals might also have reversed effects. Assuming the most popular view that NDE and NSE are hallmarks of the same and universal system of magnitude representation (sometimes even put together to constitute the numerical ratio effect) they should both be dominant, i.e., present in virtually all individuals. To the best of our knowledge, their individual prevalence has never been studied.

### Objectives of the present study

First of all, in the present study, we aim to investigate the relationship between two phenomena characterizing the analogue representation of number magnitude—namely, the NDE and the NSE—and the mathematical skills level operationalized in terms of formal education. To this end, we tested four groups of participants: professional mathematicians, engineers, social scientists, all at the level of advanced doctoral studies in their respective domain, and a reference group using the symbolic magnitude classification task with single-digit Arabic numbers. Secondly, our goal was to investigate the individual prevalence of the NDE and the NSE.

At the group level, we expected to replicate the NDE and the NSE. Taking into account that previous studies provided a somewhat inconsistent picture of relationships between the analogue numerical magnitude representations and mathematical skills, as discussed earlier, it is hard to state directional hypotheses regarding the NDE and the NSE of professional mathematicians. However, because both these phenomena are hallmarks of the analogue representation of numerical magnitude, we expected that, if they relate to mathematical skills, the direction should be the same for both NDE and NSE. In particular, the following scenarios seem possible:

- Professional mathematicians do not differ from other groups in their symbolic NDE and NSE effects. This scenario is supported by the observation that the size of the NDE does not typically account for a considerable amount of variance in mathematical skills.
- Professional mathematicians have weaker symbolic NDE and NSE than other groups, because smaller effects are typically considered to be indicators of a more precise analogue numerical magnitude representation.
- Professional mathematicians have stronger symbolic NDE and NSE compared to other groups. We do not see a strong theoretical justification for this scenario. However, mathematicians generally constitute an understudied group. Their analogue magnitude representation may be more flexible in comparison to others, leading to stronger NDE and NSE.

Regarding individual prevalence, we expect to find that the NDE and the NSE go hand-in-hand as dominant phenomena^70^, since they are both assumed to characterize the same aspect of basic symbolic numerical processing, namely an analogue magnitude representation.

## Method

### Participants

The magnitude classification task was performed by four groups of participants. There were 100 participants (47 female) in total. Their mean age was 25.2 years (SD = 3.7, range 18–35 years). All participants had normal or corrected-to-normal vision and were native Polish speakers. All participants provided informed consent, and the methods and procedures conformed to recognized ethical guidelines for testing human participants. The study was approved by the Ethics Committee for Experimental Research at the Institute of Psychology, Jagiellonian University.

Participants constituted the following groups: (1) mathematicians (henceforth M, n = 14; 2 females; mean age 28.2)—PhD studies in mathematics; (2) engineers (henceforth E; n = 15, 2 females mean age 28.1)—PhD studies in fields other than mathematics but requiring advanced math in everyday professional work (e.g., telecommunication, chemistry); (3) social scientists (henceforth S; n = 15; 2 females; mean age 27.5)—PhD studies in social sciences (i.e., psychology, sociology, philosophy, law); (4) a reference group (henceforth R; n = 56; 39 females; mean age 23.1)—individuals recruited from the general population. The inclusion criterion for the first three groups was to be at least advanced in doctoral studies (the exact dissertation topic approved by the departmental council). Although the educational background in the R group varied, nobody met the inclusion criteria for M, E, and S, nor was a PhD student nor psychology undergraduate student. The members of the groups M, E and S are the same participants as described in Cipora et al.^67^.

The large male overrepresentation in the M and the E groups reflects the general gender proportion in these cohorts. We reached out to all of the eligible PhD students and young PhDs of the Cracovian universities and tested all of those, who agreed to participate. Subsequently, we matched the gender proportion in the social sciences group (S). As the reference group (R) was initially tested in the context of another study (but using exactly the same task), and was recruited from general population, we tested all participants who signed up for the study. Given the gender proportion imbalance between the R group and M, E and S groups, in the Supplementary Material 2 we present all analyses considering male participants only and between gender comparisons regarding measures of interest to examine whether the observed differences were driven by a male advantage in the M and E groups.

The participants in the first three groups self-reported right-handedness. In the fourth group 52 participants were right-handed and 4 were left-handed. In the M, E, and S groups, the inclusion criterion was based on the writing hand. The handedness of participants in the R group is reported here based on this information as well. However, these participants also answered Oldfield’s handedness questionnaire^71^, which allows determination of handedness in a more fine-grained manner. The Oldfield questionnaire score for each participant in the R group is included in the shared data file.

### Materials

We used a computerized magnitude classification task with symbolic Arabic numbers. The participant task was to decide whether a visually presented single-digit number was smaller or larger than 5 using the Q and P keys on a standard QWERTY keyboard. Both speed and accuracy were stressed in the instruction. All stimuli were presented in black font (size 30) against a light grey background (210 210 210 in RGB notation) to avoid sharp contrasts. The task comprised two blocks with reverse response key assignment. In each block, each number (1, 2, 3, 4, 6, 7, 8, 9) was presented 30 times that gives 240 experimental trials per block. In total, 480 experimental trials of the magnitude classification task were administered to each participant. Trial order was randomized with the restriction that each number could not appear more than two times in a row. Short training sessions preceded blocks. Each training session comprised 16 trials (each number presented twice). Accuracy feedback was presented following incorrect responses, and information about response mapping was present in the bottom line of the screen. The order of blocks was counterbalanced among participants. In experimental blocks, no feedback and information about response key assignment was present. Each trial started with an eye fixation cross presented for 300 ms. Subsequently, the target number was presented until the participant responded or for a maximum duration of 2 s. The next trial started after 500 ms of blank screen presentation. A standard, MS Windows compatible computer running DMDX^72^ was used to present stimuli and collect responses. Documented experimental procedures are also shared at the Open Science Framework (https://doi.org/10.17605/OSF.IO/MSDNR).

### Procedure

The task was performed as part of a numerical cognition test battery. First of all, informed consent was obtained from all participants. Subsequently, participants sat in front of the computer and performed computerized tasks. The parity judgment task was administered first (results were reported in^67,73^; the raw data from the parity judgment task can be found at https://osf.io/tw843/). Afterwards, participants started the magnitude classification task. The magnitude classification task lasted approximately 12 min. After completion of the magnitude classification task, other tasks followed, differing between the M, E, S, and R groups. These tasks and their results are not reported here.

### Analysis

Data processing and analysis were conducted in the R language^74^. Both the data and analysis script are available at the Open Science Framework (https://doi.org/10.17605/OSF.IO/MSDNR).

To control for the stability of our data, we estimated the reliability of all effects of interest. This was done using a split-half method (Spearman-Brown corrected for double test length). A detailed description of the algorithm can be found in the Supplementary Material to Cipora et al.’s work^75^.

In the analysis, both frequentist and Bayesian approaches were used, so that we can provide evidence supporting existing effects or null effects. The NDE and the NSE were quantified by means of multiple regression analyses on RTs aggregated for each number for each participant separately. RTs were regressed on the numerical distance from the criterion value of 5 and on the numerical magnitude of numbers. Magnitude and distance predictors are orthogonal, so there is no collinearity problem. Slopes corresponding to these predictors were measures of the NDE and the NSE, respectively. A complimentary analysis considering the ratio effect is available in Supplementary Material 1. The bimanual setup with reverse response-to-key assignment allows for the measurement of the SNARC effect as well (this analysis is presented in Supplementary Material 3). In the case of numerical distance, negative slopes correspond to the typical NDE, the more negative they are, the stronger the NDE is. In the case of the NSE, positive slopes correspond to the regular size effect, and the larger they are, the stronger the effect is. To test whether the effect is present at the sample/group level, slopes were tested against 0 by means of the one-sample *t*-test (one-sided: for negative values for the NDE and positive values for the NSE). Both frequentist and Bayesian *t*-tests were used. Group comparisons were conducted by means of UNIANOVA and the BFs were computed with the anovaBF function of the R package BayesFactor^76^.

In the following step, we aimed to investigate the presence of the NDE and the NSE at the individual level. Specifically, the regression method does not allow for making inferences about the presence of effects of interest at the individual level. This is possible with a bootstrapping approach^75^. Here we adapted a H0 bootstrapping approach proposed by Cipora et al.^75^. Specifically, we aimed to check whether finding the NDE / NSE as empirically observed in each participant is likely when the null hypothesis holds, i.e. the RT pattern of a given participant does not depend on the numerical magnitude of numbers in a magnitude classification task. Therefore, separately for each participant we randomly sampled (with replacement) 8 sets of 60 trials. Subsequently, these sets were arbitrarily assigned numbers 1, 2, 3, 4, 6, 7, 8, 9 and the corresponding numerical distances from 5. These were used as predictors in a regression analysis similar to the one used to estimate the empirically observed distance and size effects. The bootstrapping procedure was repeated 5,000 times. The slopes from these bootstrap based regressions were considered as possible outcomes of the analysis if there is no NDE and no NSE. Subsequently, we checked whether empirically observed slopes were outside the mid 90% of the distribution of bootstrap slopes (i.e., the 90% H0 confidence intervals). In case of the NDE, if the empirical slope was < 0 and it was outside the 90% CI, a participant was considered to show a reliable NDE. If the slope was positive and it was outside the 90% CI, the participant was considered to show a reliable reverse NDE. If the slope was within the 90% CI, the participant was considered as not showing a reliable NDE. For the NSE, the classification is similar except that positive slopes correspond to the typical effect and negative ones to a reverse NSE.

In the last step, we compared groups with respect to the proportion of participants displaying reliable typical, non-reliable, or reliable reverse effects. (In Supplementary Material 4 we present an complete correlation matrix of all the measures we used in this study).

To check for robustness of our results, we conducted the same analysis for standardized slopes^75^. The results remained unchanged, so we do not report them in detail, however, they are available for inspection along with other shared analyses (https://doi.org/10.17605/OSF.IO/MSDNR).

### Data preprocessing

Data from two participants (one from the M group and one from the E group) were discarded from further analysis during preliminary data screening because of excessive error rates (49.5% and 50%). These participant errors can be attributed to confusion over experimental instructions (i.e., one block comprised mostly correct responses and the other mostly errors). All of the following results do not consider the data from these two individuals. Overall accuracy on the magnitude classification task was 96.9%. The ANOVA on the transformed accuracy data [2*arcsin(sqrt(proportion correct))] revealed significant between-group differences *F*(3, 94) = 3.30, *p* = 0.024, *eta*_p_^2^ = 0.095, BF = 2.21. Post hoc analysis (HSD corrected) revealed that the E group had a significantly higher performance than the R group (*p* = 0.047). However, due to very high overall performance, errors were not further analysed. Subsequently, the RT data were filtered. First, correct responses with RTs < 200 ms (0.05% of all trials) were treated as anticipations and not further analysed. Eventually, a sequential trimming method^77^ was applied: for each participant RTs outside ± 3SD from the individual mean were discarded. Ultimately, 91.1% of RT data was considered in the main analysis.

## Results

### Overview and reliability

Overall mean RT was 504 ms (SD = 79). There was no between group difference in mean RT *F*(3, 94) = 1.41, *p* = 0.246, *eta*_*p*_^2^ = 0.05, BF = 0.31. Overall mean intraindividual variability in RT was 90 ms (SD = 36). The intraindividual variability in RT did not differ between groups, *F*(3,94) = 0.52, *p* = 0.669, *eta*_*p*_^2^ = 0.02, BF = 0.13. Reliability (Split-half, Spearman-Brown corrected) of the NDE was 0.84 and reliability (Split-half, Spearman-Brown corrected) of the NSE was 0.70.

### The numerical distance effect

**Figure 1:**
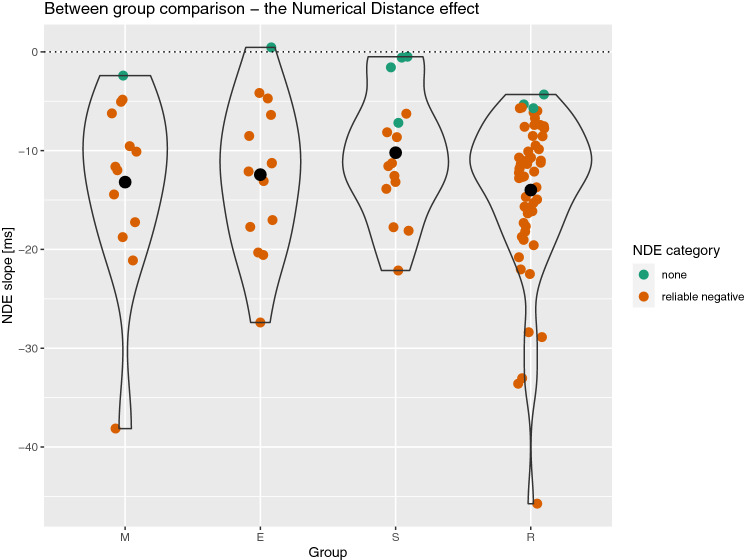
Summary of the NDE results. M = mathematicians, E = engineers, S = social scientists, R = reference group. Black dots represent group means. Coloured points (horizontally jittered) depict individual participants. Colour represents whether a given participant had a reliable NDE or not (as calculated using the bootstrapping method).

A summary of the results is presented in the left part of Table 1. The NDE was robust at the whole sample level as well as in each group, separately. This is supported by both frequentist and Bayesian analysis. However, the NDE did not differ between groups *F*(3, 94) = 0.93, *p* = 0.428, *eta*_*p*_^2^ = 0.03, BF = 0.20. The results of the bootstrapping analysis are summarized in Fig. 1. Distributions of the NDE slopes largely overlap between groups. Virtually all participants except one displayed negative slopes (indicating the typical NDE). A total of 91% of the participants had a reliable NDE. Importantly, none of the participants had a reliable reverse NDE. Proportions of participants revealing reliable slopes did not differ between groups (Fisher exact test, *p* = 0.085).

**Table 1:** The numerical distance and the numerical size effects.

Group	Numerical distance							Numerical size						
	Mean (SD)	*t*-test*	*d*	BF10	Proportion			Mean (SD)	*t*-test*	*d*	BF10	Proportion		
					Reliable (%)	Reliable reversed (%)	No reliable (%)					Reliable (%)	Reliable reversed (%)	Not reliable (%)
Overall	− 13.08 (7.90)	***t*(97)** **=** **−** **16.39,** ***p*** **<** 0.001**	1.66	> 10^26^	91	0	9	1.20 (3.16)	***t*(97)** **=** **3.77,** ***p*** **<** **0.001**	0.38	142.00	29	11	60
M	− 13.19 (9.41)	***t*(12)** **=** **−** **5.06,** ***p*** **<** **0.001**	1.40	234.00	92	0	8	0.81 (3.20)	*t*(12) = 0.92, *p* = 0.189	0.25	0.63	38	15	46
E	− 12.43 (7.60)	***t*(13)** **=** **−** **6.12,** ***p*** **<** **0.001**	1.64	1,391.00	93	0	7	1.68 (3.19)	***t*(13)** **=** **1.97,** ***p*** **=** **0.035**	0.53	2.31	29	14	57
S	− 10.21 (6.47)	***t*(14)** **=** **−** **6.12,** ***p*** **<** **0.001**	1.58	1846.00	73	0	27	0.37 (2.06)	*t*(14) = 0.69, *p* = 0.250	0.18	0.48	27	20	53
R	− 13.98 (7.96)	***t*(55)** **=** **−** **13.15,** ***p*** **<** **0.001**	1.76	> 10^15^	95	0	5	1.40 (3.39)	***t*(55)** **=** **3.08,** ***p*** **=** **0.002**	0.41	19.10	27	7	66

*One sample *t*-test against zero (one sided); **significant results are marked with a bold font; *M* mathematicians, *E* engineers, *S* social scientists, *R* reference group.

### The numerical size effect

**Figure 2:**
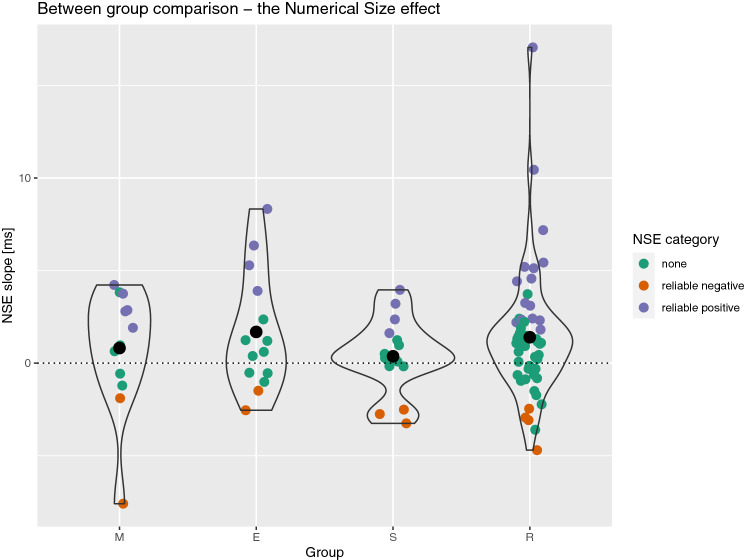
Summary of the NSE results. M = mathematicians, E = engineers, S = social scientists, R = reference group. Black dots represent group means. Coloured points (horizontally jittered) depict individual participants. Colour represents whether the given participant had a reliable size effect, a reliable reverse size effect, or no reliable size effect (as calculated using the bootstrapping method).

A summary of the results is presented in the right part of Table 1. The NSE was robust at the whole sample level as well as in the R and the E group. It did not reach significance in any other group. However, in the M, E, and S groups Bayesian evidence was highly inconclusive. Again, groups did not differ regarding the NSE, *F*(3, 94) = 0.59, *p* = 0.626, *eta*_*p*_^2^ = 0.02, BF = 0.13. The results of the bootstrapping analysis are summarized in Fig. 2. Interestingly, only 29% of the participants displayed a reliable NSE. On the other hand, 11% of the participants had a reliable reverse NSE. Most importantly, 60% of participants did not display a reliable NSE. The proportions of participants displaying reliable, reliable reverse, and no NSE did not differ between groups (Fisher exact test *p* = 0.629).

## Discussion

### Overview

This study aimed to investigate how two hallmark effects of the analogue representation of symbolic numerical magnitude, namely the NDE and the NSE, relate to mathematical skills. In particular, we were interested in these phenomena at a very high level of mathematical skills operationalized in terms of formal education. For this purpose, we recruited four groups of participants: professional mathematicians, engineers, social scientists, and a reference group. We administered the magnitude classification task with single-digit Arabic numbers. Secondly, we checked the individual prevalence of the phenomena of interest, i.e., how many individuals reveal a reliable NDE and NSE.

We did not find between group differences, despite replicating a robust group level NDE both at the whole sample level and in each group separately. Bayesian analysis provided direct support for no between group differences in NDE. The NSE was also robust at the whole sample level, in the reference group and in the engineers group, but it did not reach significance in any other group. On the other hand, again, groups did not differ with respect to the NSE, which was also supported by Bayesian evidence. Analysis of individual prevalence also did not reveal any between group differences as regards both phenomena under scrutiny. Notably, the lack of between group differences cannot be accounted for by unequal proportions of male and female participants: there was no overall gender difference as regards the NDE and the NSE, and the above patterns of results were similar when only male participants were analysed (see Supplementary Material 2).

Nevertheless, the analysis of individual prevalence provided insight into general properties of the NDE and NSE. The NDE seems to be reliably present in virtually all participants, and none of the participants revealed a reliable reverse NDE. The results pattern for the NSE was different. There were several individuals who had a reliable reverse NSE, but more than 60% of the participants did not display either a reverse or a typical NSE.

### Analogue magnitude processing of symbolic numbers in mathematicians: The numerical distance and size effects

Taking into account that due to intense training and daily exposure to symbolic numbers, professional mathematicians might be expected to differ in their ability to access number magnitude information (or that the specific type of analogue representation has helped them to become professional mathematicians). On the other hand, previous studies suggest that the size of the NDE remains unchanged until adulthood^20,21^, and it presumably does not depend on the mathematical skills level^58^, at least when groups without learning problems are considered. Our study generally aligns with this pattern of results. Notably, even when groups with mathematical difficulties are taken into account, available evidence together suggests no differences in the measured NDE.

Regarding the NSE, the only intergroup differences reported up to date concern individuals with mathematical learning problems and math-anxiety. Reports on the relationship between the level of mathematical competence and the NSE are not known to us. Rousselle and Noël^64^ found that mathematical learning problems go hand in hand with a reduced NSE, while Núñez-Peña and Suárez-Pellicioni^69^ showed a correlation between high levels of math-anxiety and a larger NSE. Although the results of Rousselle and Noël are hard to elucidate theoretically, Núñez-Peña and Suárez-Pellicioni suggest that highly math-anxious individuals have a less precise access to numerical magnitude.

In our study we found no differences between mathematicians and other groups. Importantly, the same pattern of results remained unchanged irrespective of the approach we used. It held both for unstandardized and standardized NDE and NSE slopes, as well as when we took into account proportions of participants revealing reliable effects.

### What constitutes extremely high mathematical skills level?

Testing extreme groups within a given domain has provided instructive insights in several fields of psychology and cognitive science^78^. Extreme groups may reveal effects that are blurred in typical level groups (e.g., due to limited variance). Also, the field of numerical cognition has gained valuable insights by testing extreme groups in terms of mathematical skills. Investigations on mathematical cognition have mostly been focused on groups displaying mathematical difficulties^79–81^. Studies performed on groups displaying high mathematical skills levels are rare, and “high-level math ability” has been inconsistently defined; sometimes as prodigious calculators or sometimes as professional mathematicians. Prodigious calculators are individuals who perform complex arithmetic tasks very fast and efficiently^82^. Nevertheless, their skills are usually limited to a set of arithmetic problems and originate mostly from extended drill^19^. On the other hand, academic mathematical expertise is typically understood as being able to swiftly operate on mathematical theorems, concepts, conduct rigorous proofs, and discover new mathematical laws in a creative way^83–85^, which may doubly dissociate from calculation proficiency^86^.

Prodigious calculators described in the literature can hardly be considered to show extremely high levels of mathematical skills. Mathematical education does not solely aim at excelling in mental calculation procedures, it instead aims at an increased understanding of mathematical concepts and operating on them, aspects typically not mastered by prodigious calculators^86^. This means that previous research showing the NDE in a calculation prodigy^66^ cannot be generalized to professional mathematicians. Although many fields of professional practice, like engineering, require familiarity with advanced mathematical tools, individuals involved in them share the above characteristics of professional mathematical activity very rarely. As in our previous study^67^, here, we considered engineers and mathematicians as separate groups.

Despite the fact that the existing knowledge-base is quite narrow, previous studies revealed that professional mathematicians differ from non-mathematicians in several cognitive aspects. Although there is no space here to review all of them, these differences result from a configuration of domain-general factors such as fluid intelligence^67^, arithmetic operations skills^68,87–89^, and spatial-numerical mappings^67,68^. Analogue processing of symbolic numerical magnitudes constitutes the understudied category of basic numerical skills of professional mathematicians. It is worth noting that this category should be considered as distinct from spatial-numerical associations^90^.

### The construct validity of the common magnitude system for symbolic number magnitude effects

The correlation between the NDE and the NSE was low, even when adjusting for non-perfect task reliabilities (Supplementary Material 4). Importantly, the correlation vanished when we controlled for overall RT. This finding is somehow surprising since both phenomena are considered to be manifestations of an analogue magnitude representation^19,32^. Usually in diagnostics, for measures taken to indexing the same underlying construct, one would expect and require high intercorrelations of related measures. It is important to note that the reliability of our measures was still satisfactory, so that reliability issues cannot account for the low correlation and not at all, for the null correlation, when overall RT is partialled out. For these reasons, these results challenge the construct validity of a common magnitude system like the ANS for the case of symbolic number magnitude processing.

On the other hand, our findings can be accounted for by another framework for magnitude processing, namely Krajcsi’s DSS framework^34,91,92^. It postulates that the symbolic NDE and NSE emerge from two independent mechanisms. The DSS stores symbolic numbers in an amodal way as nodes within a conceptual network. Numerical properties are encoded as the connections between these nodes. The NDE reflects the semantic distance between the numbers, while the NSE appears as the consequence of the frequency of symbols, i.e., the fact that larger numbers are typically less frequent than smaller ones in daily life^93^. On the other hand, non-symbolic comparative judgements could still be processed by the ANS. In contrast to the ANS framework that assumes a strong correlation between the symbolic NDE and NSE, within the DSS framework, they could be partly dissociated. Indeed, Krajcsi^91^ found no correlation between the NDE and the NSE when participants processed symbolic numbers. Our results corroborate this pattern and could be interpreted in a line with the DSS framework of human magnitude representation of symbolic numbers.

### The individual prevalence of symbolic numerical distance and size effects

We found a reliable NDE for almost all participants. On the other hand, prevalence of the NSE was much lower. Only 29% of participants displayed a reliable NSE, a reverse reliable NSE occurred for about 11% of the individuals, while 60% did not have a reliable NSE.

Using the recent distinction of psychological phenomena into dominant and indominant ones^70^, we can conclude that although the NDE is a dominant phenomenon occurring in all individuals (like the Stroop effect), and the reverse effect is not observed at all, the NSE seems to be indominant. In the field of numerical cognition a pattern similar to the NSE was recently observed for the SNARC effect that appears to be reliably present only in about 45% of individuals^75^. Note, however, that these phenomena belong to different categories of basic numerical cognition: the NSE and the NDE do not have a spatial component^42,43^. The dissociation we found calls for future research, because it seems to be at odds with the most common interpretation of the NDE and the NSE.

### Limitations of the study and future research directions

In this study, we used the symbolic magnitude classification task with single-digit Arabic numbers. There are at least two limits of our investigation. The first one concerns the relative ease of the task. The goal of this study was to examine whether professional mathematicians do already differ in basic numerical effects, which are supposed to index basic numerical representations like the symbolic magnitude representation. To best our knowledge, no one has tested before the hallmark effect of the symbolic magnitude processing, the symbolic numerical distance effect in professional mathematicians. Since the symbolic numerical distance effect is observed in the overwhelming majority of studies with single-digit numbers, we decided to use single-digit stimuli. We believe that our approach is simple but not too simple since we found stable and robust effects of interest in all tested groups. Undoubtedly, further research should test whether professional mathematicians differ from other populations in the processing of more complex, i.e., multi-digit, numerical material. We hypothesize that using two-digit numbers could change the pattern of results, especially reveal differences in the NDE and NSE between mathematicians and other groups, because they are more complex. Especially, place-value processing as an additional process that is automatically initiated, whenever two-digit numbers are processed^94^. However, we claim that presumed differences would not only be due to the numerical difficulty level of the task (larger number size, less frequent numbers, additional place-value processing), but other factors producing considerable differences in single-digit and multi-digit number processing as the involvement of strategies and domain general-processes^38–41^. To sum up, our data should be interpreted with care. The fact that professional mathematicians do not differ from controls in the most basic tasks and effects of symbolic numerical magnitude processing does not imply that there will also be null differences in more complex stimuli or tasks.

The second limitation concerns a non-symbolic dimension of the analogue number magnitude representation. Although we are aware that a link between the non-symbolic magnitude processing and mathematical skills is well documented^48,52,55,95^, here we tested all the groups using only the symbolic magnitude classification task since the symbolic number processing accounts the more variance in mathematical skills^53,54^. A well-established tradition of mathematical cognition research, from Moyer and Landauer’s study^4^, through Dehaene’s triple code framework^1^, to more recent computational model^35^, accounts the processing of both symbolic and non-symbolic numerical magnitudes within the single analogue cognitive system usually called ANS^19,32^. Moreover, the shared behavioural patterns in the analogue magnitude processing, including all the numerical^4–8^, and non-numerical instances^9–16^ could be accounted within a unified ATOM framework^17^. These frameworks focus on similarities, but this does not exclude the existence of differences within the magnitude system depending on the task. This is substantiated in the fact that the symbolic NDE is uncorrelated with the non-symbolic NDE^96^. Moreover, Krajcsi’s framework discussed above assumes that symbolic and non-symbolic numerical magnitudes are handled by independent cognitive systems (namely non-symbolic by ANS and symbolic by DSS)^34,91^. Therefore, a generalization of the behavioural pattern we found in the symbolic processing domain to the non-symbolic one would be unsound. Regardless of the applied theoretical framework, differences between professional mathematicians and other groups in analogue magnitude tasks involving non-symbolic numbers (and non-numerical material) could exist and should be accounted for in subsequent studies.

## Conclusions

Analogue magnitude is one of multiple mental representations of symbolic numbers, and the NDE and the NSE constitute its primary instances. Even though these phenomena were previously revealed in various human cultures and age groups, as well as in non-human animals, there is no consensus about their relationship to mathematical skills. Furthermore, so far, studies on the NDE and the NSE have been carried out at the group level, with nothing known about their individual prevalence. Testing professional mathematicians, engineers, social scientists, and the reference group, we found no between group differences in the NSE and the NDE. This observation allows us to infer that the professional training and practice of mathematicians does not change their analogue magnitude representation of numbers or alternatively, that possessing a specific type of magnitude representation does not foster mastering professional mathematical skills. Looking at the prevalence of the phenomena for single-digit numbers at the individual level, we found a reliable NDE in almost all participants, whilst the prevalence of a reliable NSE was surprisingly much lower. This indicates that the former effect is dominant, whereas the latter is indominant. This last conclusion especially calls for further research on whether NDE and NSE truly reflect properties of the same system of representing magnitude, which is assumed to be universal in all humans. Importantly, this conclusion cannot be accounted for by the fact that NSE was just weaker than the NDE (e.g., because we used single-digit numbers only): when controlling for the mean RT, NDE and NSE did not correlate with each other (see Table S2 in Supplementary Material 4).

## Supplementary information

Supplementary information

## Data Availability

Experimental task used in the study as well as both the data and analysis script are available at the Open Science Framework (https://doi.org/10.17605/OSF.IO/MSDNR).
