# Supplementary material for: Professional mathematicians do not differ from others in the symbolic numerical distance and size effects
Source: Sci Rep. 2020 Jul 13;10:11531. doi: 10.1038/s41598-020-68202-z (PMC7359336; doi:10.1038/s41598-020-68202-z)
Supplement: Supplementary file 1 — Supplementary information [file 41598_2020_68202_MOESM1_ESM.docx]

**Supplementary Materials to:**

**Professional mathematicians do not differ from others in the symbolic numerical distance and size effects**

Mateusz Hohol, Klaus Willmes, Edward Nęcka, Bartosz Brożek,

Hans-Christoph Nuerk & Krzysztof Cipora

**Supplementary Material 1:** **The ratio effect**

In an additional analysis, we checked whether our conclusions regarding between group differences hold, if instead of the NDE and the NSE we consider the numerical ratio^1^. Scripts used to conduct this analysis are available online via Open Science Framework (<https://doi.org/10.17605/OSF.IO/MSDNR>).

In this analysis, we again used individual regression slopes, in which we regressed mean RTs on the numerical ratio. The ratio was calculated for each number in the set separately. It was the numerical ratio of the number being presented and number 5. To obtain the ratios, for each number and the criterion number 5, we divided the smaller number by the larger one (i.e. numbers 1-4 were divided by 5, while 5 was divided by numbers 6-9). The ratios were .2, .4, .6, .8, .83, .71, .63, and .56 for numbers 1, 2, 3, 4, 6, 7, 8, 9 respectively. A more positive slope indicates a stronger ratio effect. Noteworthy, the ratio effect combines the NDE and the NSE into a single measure. Importantly, the numerical distance influences the numerical ratio effect more than the absolute magnitude of the numbers. In case of our stimulus set, the NDE correlated with the ratio at -.83, while the magnitude of the number to be compared with 5 correlated at .55. As both absolute size and numerical distance are used for calculating the ratio, such an analysis does not allow disentangling their relative influence.

The reliability of the ratio effect (split-half, Spearman-Brown corrected) was .85. The ratio effect was robust at the whole sample level, *t*(97) = 14.70, *p* < .001, *d* = 1.48, BF_10_ > 10^23^. The ratio effect was also robust in each group (all *p*s < .001, *d*s ≥ 1.45, BF_10_s ≥ 228). Again, groups did not differ regarding the ratio effect, *F*(3,94) = 1.11, *p* = .351, *eta_p_*^2^ = .03. Bayesian analysis provided evidence for the lack of between group differences, BF_10_ = -.24.

Bootstrapping revealed that the vast majority (88%) of the participants showed a reliable ratio effect. Notably, none of the participants was found with a reverse ratio effect (cf. Figure S1). Groups did not differ in the proportion of participants revealing a reliable ratio slope (Fisher exact test *p* = .565).

The results do not change when only male participants in all groups are considered.


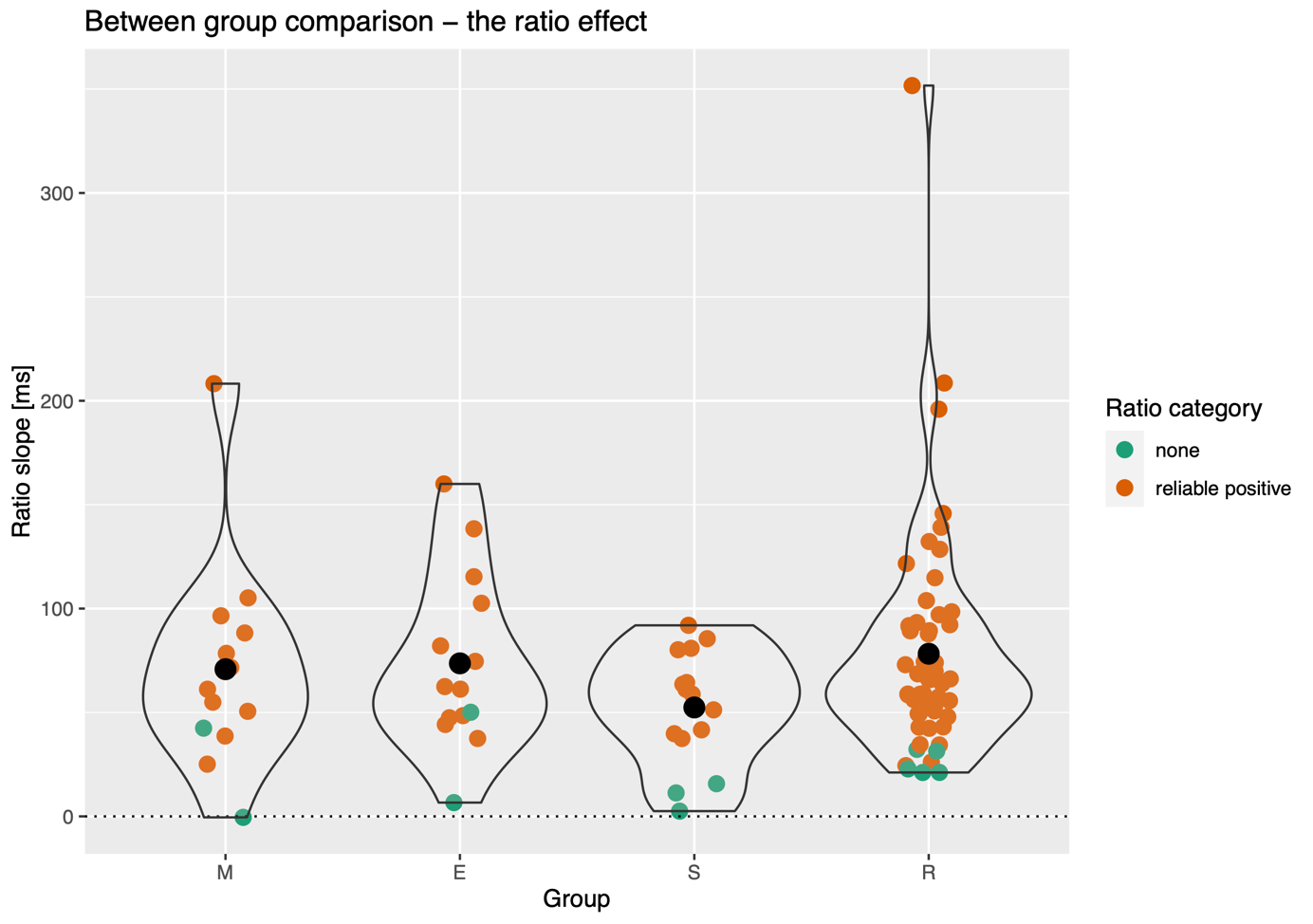


*Figure S1*. Summary of the ratio effect results. Black dots represent the respective group means. Colored points (horizontally jittered) depict individual participants. Color represents whether the given participant reveals a reliable ratio effect or no reliable ratio effect, as calculated using the bootstrapping method.

To sum up, the main conclusion of the paper, that there are no between group differences in symbolic numerical distance and size effects holds as well when the ratio effect comprising both these phenomena is considered. Thus, this extra analysis provides an additional robustness check for our conclusion.

**Supplementary material 2:** **Gender differences and analysis considering male participants only**

As rightfully pointed by one of the reviewers, the groups were not gender balanced. The reason for that is that when we were recruiting M and E groups, we could mostly recruit male participants. The gender proportion in our sample at least to some extent reflects the gender proportion of PhD students in mathematics and engineering faculties, at least at universities, from which we recruited our participants. The gender proportion in the S group was matched to the M and E groups. At the same time, the R group was recruited from the general population.

To the best of our knowledge, there is only one study investigating gender differences in the NDE (see Experiment 4 in^2^). This study found a gender effect (males not revealing the NDE at all). However, the task differed considerably from our setup (it utilized a dual task with tone discrimination) and probably for this reason the reported reaction times for all distances exceeded 1000ms. We are not aware of any data on gender differences regarding the NSE.

However, to check whether the lack of between group differences originates from confounds caused by a potential gender difference, we first calculated gender differences across all participants and in the R group (a gender comparison would not be possible due to the very small number of female participants in the M, E, and S groups). Subsequently, we analyze the data from male participants only.

**Gender differences**

Neither at the whole sample level nor in the R group there were any gender differences in the NDE and the NSE (all *p*s > .28, all *d*s < 0.31, BF_10_s < 0.48).

**Analysis considering male participants only**

An additional analysis considered male participants only (overall *n* = 53, 11, 12, 13, and 17 participants in the M, E, S, and R groups, respectively). Here we present only a summary of the results; all results and analyses can be obtained by running the shared analysis codes. Please note that this analysis considers even smaller samples in the M, E, and S groups, so they should be treated with caution.

*Overall performance*

There was a significant difference in overall accuracy between groups, *F*(3,49) = 6.45, *p* < .001, *eta_p_*^2^ = .28, BF_10_ = 43.01. The effect was driven by the R group, which performed significantly worse than the E group and the M group (*p*s .001 and .011 respectively, HSD corrected). The difference was more pronounced when males only were analyzed, and in contrast to main analysis, the R group differed not only form the E group but also from the M group.

Similarly to the main analysis, groups did not differ in mean RTs, *F*(3,49) = 0.64, *p* = .596, *eta_p_*^2^ = .04, BF_10_ = 0.18. There was also no difference in intraindividual variability in RTs, *F*(3,49) = 0.16 *p* = .925, *eta_p_*^2^ = .01, BF_10_ = 0.11.

*Numerical distance and size effects*

Reliability of the effects was very similar to the main analysis (.88 for NDE and .74 for NSE). Again, the results remained unchanged when we considered standardized rather than unstandardized slopes.

*Numerical distance effect*

As in the main analysis, the NDE was robust at the whole sample level and in all subgroups (cf. Table S1). There was also no between group difference in the NDE, *F*(3,49) = 0.13, *p* = .934, *eta_p_*^2^ = .01, BF_10_ = 0.12.

*Numerical size effect*

As in the main analysis, the NSE was robust at the whole sample level (cf. Table S1. Similar to the main analysis, the effect was significant in the E group. Contrary to the main analysis, the effect was not significant in the R group any more. As in the main analysis, the NSE was not significant in the M and the S group. In the case of all these group level effects (and lacks thereof), the BFs remained inconclusive. Similar to the main analysis, there were no between group differences in the NSE, *F*(3,49) = 0.40, *p* = .756, *eta_p_*^2^ = .02, BF_10_ = 0.15.

*Prevalence*

As in the main analysis, there were no between group differences in the prevalence of reliable NDE (*p* = .919) and NSE (*p* = .594).

*Correlations between phenomena*

The correlation between NDE and NSE was -.32. Again, this correlation was no longer present when controlling for mean RT.

*Table S1*. The numerical distance and the numerical size effects – only male participants considered.

| Group | Numerical distance | | | | | | | Numerical size | | | | | | |
| --- | --- | --- | --- | --- | --- | --- | --- | --- | --- | --- | --- | --- | --- | --- |
|  | Mean (SD) | *t*-test* | *d* | BF10 | Proportion | | | Mean (SD) | *t*-test* | *d* | BF10 | Proportion | | |
|  |  |  |  |  | Reliable | Reliable reversed | No reliable |  |  |  |  | Reliable | Reliable reversed | No reliable |
| Overall | -12.50 (8.25) | ***t*(52) = -11.00, *p* < .001**** | 1.52 | > 10^12^ | 91% | 0% | 9% | 1.10 (3.47) | ***t*(52) = 2.31, *p* = .012** | 0.32 | 3.35 | 26% | 17% | 57% |
| M | -13.80 (9.95) | ***t*(10) = -4.65, *p* < .001** | 1.39 | 82.80 | 91% | 0% | 9% | 0.76 (3.42) | *t*(10) = 0.74, *p* = .240 | 0.22 | 0.56 | 36% | 18% | 45% |
| E | -12.7 (8.17) | ***t*(11) = -5.39, *p* < .001** | 1.56 | 291.00 | 92% | 0% | 8% | 1.81 (3.40) | ***t*(11) = 1.97, *p* = .046** | 0.53 | 1.97 | 33% | 17% | 50% |
| S | -11.60 (5.70) | ***t*(12) = -7.35, *p* < .001** | 2.04 | 4885.00 | 85% | 0% | 15% | 0.40 (2.22) | *t*(12) = 0.65, *p* = .264 | 0.18 | 0.48 | 31% | 23% | 46% |
| R | -12.20 (9.33) | ***t*(16) = -5.40, *p* < .001** | 1.31 | 907.00 | 94% | 0% | 6% | 1.36 (4.39) | *t*(16) = 1.28, *p* = .109 | 0.31 | 0.88 | 12% | 12% | 76% |

*Notes.* * one sample *t*-test against zero (one sided); ** Significant results are marked with a bold font; *** M = mathematicians, E = engineers, S = social scientists, R = reference group.

**Supplementary Material 3: The SNARC effect**

*Analysis*

The analysis reflects the approach reported in the main text. To quantify the SNARC effect^3^, we used the individual regression slopes (just as for the distance and the size effects described in the main text). Importantly, the SNARC effect in the magnitude classification task, contrary to the parity judgment task, is categorical rather than linear^4,5^. Therefore, we quantified the SNARC effect as the individual regression slope, where dRTs (RT differences: right hand – left hand) are regressed on a magnitude contrast: numbers smaller than 5 were coded as -.5 and numbers larger than 5, as +.5. More negative slopes correspond to a stronger SNARC effect. First, we estimated the reliability of the SNARC effect slopes; subsequently, using one-sample *t*-tests (one-sided), we tested for the presence of the SNARC effect at the whole sample level as well as in each of the four groups separately; we also compared groups by means of ANOVA as regarded the size of the slope. As in the main analysis, both frequentist and Bayesian analyses were used. In the next step, we tested for the individual prevalence of the SNARC effect using the H0 bootstrapping approach. We resampled RTs for each number (with replacement) and randomly allocated them into two subsets, which were subsequently treated as Right-hand and Left-hand responses. As in the analyses reported in the main text, 90% H0 CIs were used, and participants were classified based on whether they displayed a reliable SNARC effect. Finally, by means of a Fisher exact test, we tested for differences in the proportion of participants displaying a reliable SNARC effect, a reliable reverse SNARC effect, or no reliable SNARC effect.

*Testing for the SNARC effect*

The reliability (split-half, Spearman-Brown corrected) estimate of the SNARC slopes was .89. The SNARC slopes at the whole sample level as well as in each group separately are presented in Table S2. The SNARC effect was robust at the whole sample level as well as in the R and E groups. On the other hand, the effect was not significant either in the M or the S group. In all the groups except the R group, Bayesian evidence was inconclusive and did not provide much support in favor of either the null or the alternative hypothesis. However, groups did not differ with respect to the SNARC effect as indicated by both frequentist and Bayesian evidence: *F*(3, 94) = 1.37, *p* = .257, *eta_p_*^2^ = .04, BF_10_ = 0.29.

*Table S2.* The SNARC effect at the whole sample level and in each group separately.

| Group | Mean (SD) | t-test* | *d* | BF 10 | Proportion | | |
| --- | --- | --- | --- | --- | --- | --- | --- |
|  |  |  |  |  | Reliable SNARC | Reliable reverse SNARC | No reliable SNARC |
| Overall | -16.0 (50.6) | ***t*(97) = -3.14, *p* = .001**** | 0.32 | 21.60 | 42% | 16% | 42% |
| M | 8.1 (52.6) | *t*(12) = 0.56, *p* = .706 | 0.16 | 0.20 | 31% | 23% | 46% |
| E | -24.4 (48.4) | ***t*(13) = -1.88, *p* = .041** | 0.50 | 2.05 | 50% | 21% | 29% |
| S | -10.6 (50.2) | *t*(14) = -0.82, *p* = .212 | 0.21 | 0.55 | 47% | 20% | 33% |
| R | -21. (50.2) | ***t*(55) = -3.13, *p* = .001** | 0.42 | 21.90 | 41% | 13% | 46% |

*Notes.* * one sample *t*-test against zero (one sided); ** Significant results are marked with a bold font; *** M = mathematicians; E = engineers; S = social scientists; R = reference group.

The results of the bootstrapping analysis are summarized in Figure S2. The distributions of SNARC effect slopes largely overlap between groups. Interestingly, only 41.8% of participants had a reliable SNARC effect. On the other hand, 16.3% of participants displayed a reliable reverse SNARC effect. Most importantly, 41.8% of participants did not have a reliable SNARC effect. The proportions of participants displaying a reliable, a reliable reverse, or no reliable SNARC effect did not differ between groups (Fisher exact test *p* = .739).


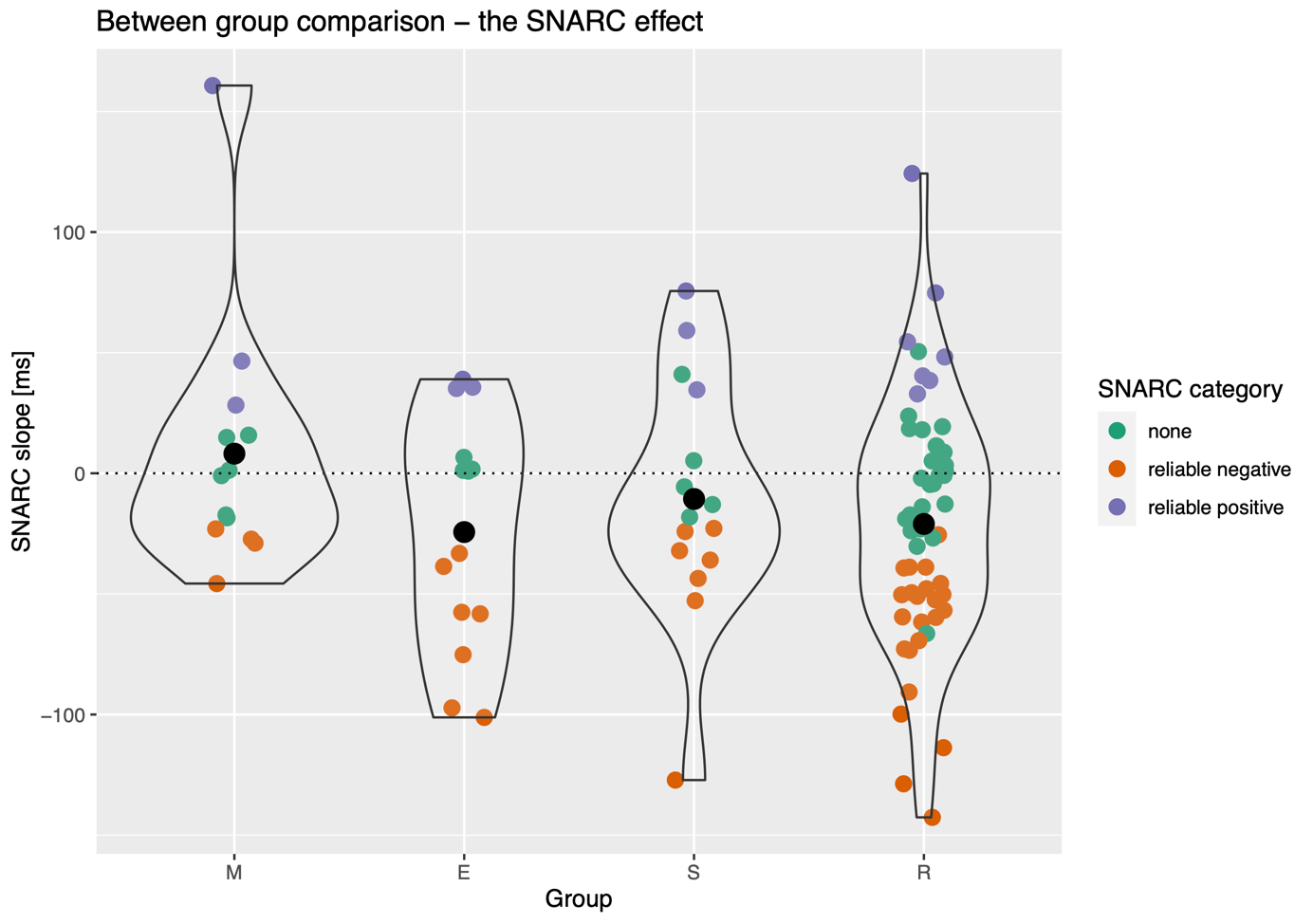


*Figure S2.* Summary of the SNARC effect results. M = mathematicians, E = engineers, S = social scientists, R = reference group. Black dots represent the respective group means. Colored points (horizontally jittered) depict individual participants. Color represents whether the given participant reveals a reliable SNARC effect, a reliable reverse SNARC effect, or no reliable SNARC effect, as calculated using the bootstrapping method.

**Gender effects**

*Between gender comparison*

At the whole sample level there was no gender difference in the SNARC effect, *t*(96) = 1.24, *p* = .220, *d* = 0.25, BF_10_ = 0.42. When only the R group was considered, the effect reached significance, *t*(54) = 2.01, *p* = .050, *d* = 0.58, BF_10_ = 1.45. Males tended to reveal a stronger SNARC effect (-40.87, *SD* = 36.11) than females (-12.35, *SD* = 53.37). However, as indicated by Bayesian statistics, this result remains largely inconclusive.

*Analysis of the SNARC effect considering male participants only*

When data from male participants only were analyzed, the results remained largely unchanged. The reliability of the SNARC effect was high, and it was robust at the whole sample level as well as in the E and the R group (along with conclusive BFs). At the same time, it was not significant in the M or the S groups (BFs inconclusive). Contrary to the main analysis, there was a significant between group difference in the SNARC effect, *F*_3,49_ = 3.43, *p* = .024, *eta_p_*^2^ = .17. However, Bayesian evidence remained inconclusive (BF_10_ = 2.48). Post hoc analysis (HSD) indicated that this effect was driven by the difference between the M and the R group (*p* = .022). There was no between group difference in the prevalence of a reliable SNARC effect.

**Supplementary Material 4:** **Correlations**

*Table S3.* Zero order correlations between measures used in the study.

| Measure | | Accuracy | RT | SD(RT) | Distance | Size | SNARC |
| --- | --- | --- | --- | --- | --- | --- | --- |
| Accuracy | *r* | — | **.29** | .06 | **-.28** | -.01 | **.27** |
|  | *p* | — | **.004** | .579 | **.006** | .937 | **.008** |
|  | 95% CI Upper | — | **.46** | .25 | **-.08** | .19 | **.44** |
|  | 95% CI Lower | — | **.09** | -.14 | **-.45** | -.21 | **.07** |
| RT | *r* |  | — | **.90** | **-.71** | **.41** | .04 |
|  | *p* |  | — | **< .001** | **< .001** | **< .001** | .667 |
|  | 95% CI Upper |  | — | **.93** | **-.60** | **.56** | .24 |
|  | 95% CI Lower |  | — | **.86** | **-.80** | **.23** | -.16 |
| SD(RT) | *r* |  |  | — | **-.71** | **.52** | -.01 |
|  | *p* |  |  | — | **< .001** | **< .001** | .943 |
|  | 95% CI Upper |  |  | — | **-.59** | **.65** | .19 |
|  | 95% CI Lower |  |  | — | **-.79** | **.36** | -.21 |
| Distance | *r* |  |  |  | — | **-.25** | -.05 |
|  | *p* |  |  |  | — | **.012** | .602 |
|  | 95% CI Upper |  |  |  | — | **-.06** | .15 |
|  | 95% CI Lower |  |  |  | — | **-.43** | -.25 |
| Size | *r* |  |  |  |  | — | -.15 |
|  | *p* |  |  |  |  | — | .146 |
|  | 95% CI Upper |  |  |  |  | — | .05 |
|  | 95% CI Lower |  |  |  |  | — | -.34 |
| SNARC | *r* |  |  |  |  |  | — |
|  | *p* |  |  |  |  |  | — |
|  | 95% CI Upper |  |  |  |  |  | — |
|  | 95% CI Lower |  |  |  |  |  | — |

*Note:* Significant correlations are marked with bold font.

*Table S4.* Partial correlations between the NDE, NSE, and the SNARC effect controlled for mean reaction time.

| Measure | Distance | Size | SNARC |
| --- | --- | --- | --- |
| Distance | - | 0.06 | -0.03 |
| Size |  | - | -0.18 |

*Note:* None of the partial correlations was significantly different from zero.

**References**

1. Lyons, I. M., Nuerk, H.-C. & Ansari, D. Rethinking the implications of numerical ratio effects for understanding the development of representational precision and numerical processing across formats. *J. Exp. Psychol. Gen.* **144**, 1021–1035 (2015).

2. Bull, R., Cleland, A. A. & Mitchell, T. Sex differences in the spatial representation of number. *J. Exp. Psychol. Gen.* **142**, 181–192 (2013).

3. Dehaene, S., Bossini, S. & Giraux, P. The mental representation of parity and number magnitude. *J. Exp. Psychol. Gen.* **122**, 371–396 (1993).

4. Gevers, W., Verguts, T., Reynvoet, B., Caessens, B. & Fias, W. Numbers and space: A computational model of the SNARC effect. *J. Exp. Psychol. Hum. Percept. Perform.* **32**, 32–44 (2006).

5. Wood, G., Willmes, K., Nuerk, H.-C. & Fischer, M. H. On the cognitive link between space and number: A meta-analysis of the SNARC effect. *Psychol. Sci. Q.* **50**, 489–525 (2008).
